# Anatomy education in US Medical Schools: before, during, and beyond COVID-19

**DOI:** 10.1186/s12909-022-03177-1

**Published:** 2022-02-16

**Authors:** Max Shin, Aman Prasad, Graham Sabo, Alexander S. R. Macnow, Neil P. Sheth, Michael B. Cross, Ajay Premkumar

**Affiliations:** 1 Perelman School of Medicine, University of Pennsylvania, Philadelphia, PA 19104 USA; 2 Cornell University, Ithaca, NY 14853 USA; 3 Foundation for Advancement of International Medical Education and Research, Philadelphia, PA 19104 USA; 4 Department of Orthopaedic Surgery, University of Pennsylvania, Philadelphia, PA 19104 USA; 5 Department of Orthopaedic Surgery, Hospital for Special Surgery, New York, NY 10021 USA

**Keywords:** Anatomy, COVID-19, Technology

## Abstract

**Background:**

Anatomy education in US medical schools has seen numerous changes since the call for medical education reform in 2010. The purpose of this study was to survey US medical schools to assess recent trends in anatomy education, the impact of the COVID-19 pandemic on anatomy teaching, and future directions of medical school anatomy curricula.

**Methods:**

We sent a 29-item survey to anatomy course directors of 145 AAMC-associated allopathic medical schools inquiring about their schools’ anatomy curricula. The survey contained objective discrete questions concerning the curricula changes preceding COVID-19 and those directly related to COVID-19. We also asked subjective and open-ended questions about the impact of COVID-19 and future directions of anatomy education.

**Results:**

A total of 117/143 course directors (82%) completed the survey. Most schools (60%) reported a major change to their anatomy course within the past five years, including a decrease in total course time (20%), integration of anatomy into other courses (19%), and implementation of a “flipped classroom” (15%) teaching style. Due to COVID-19, there was a decrease in the fraction of course time dedicated to “hands-on” learning (*p* < 0.01) and teaching of clinical correlates (*p* = 0.02) and radiology (*p* < 0.01). Most course directors (79%) reported that COVID-19 had a negative impact on quality of learning due to decreased interactive or in-person (62%) learning and lack of dissection (44%). Incorporation of virtual-reality applications or 3D anatomy software (23%) and a decrease in cadaver dissection (13%) were the most common future anticipated changes.

**Conclusion:**

The constraints conferred by COVID-19 highlight the importance of maximizing interactive learning in the discipline of anatomy. In an era of social distancing and decreased emphasis on conventional anatomy dissection, adaptations of new technologies and teaching modalities may allow for traditional educational rigor to be sustained.

**Supplementary Information:**

The online version contains supplementary material available at 10.1186/s12909-022-03177-1.

## Introduction

Over the past decade, the landscape of United States (US) medical education has continuously changed following calls to adopt innovative, competency-based curricula to produce physicians better prepared to navigate our complex health care system [1]. Notable changes have included adoption of new technologies, a greater emphasis on team-based learning, enhancement of interprofessional education, and condensation of the preclinical curriculum. In particular, numerous institutions in the US have recently compressed their basic sciences or foundational preclinical curricula from the traditional 24 months to 12 or 18 months [2, 3]. Furthermore, the COVID-19 pandemic has had significant impacted all facets of medical education, requiring physician educators to redesign curricula to be in line with social distancing mandates. For many preclinical courses, the aforementioned changes may have simply entailed reduced formalized didactics, more case-based modules, and a transition to online, recorded lectures. However, such modifications would be more difficult for subjects with a physical laboratory component, such as gross anatomy, which has conventionally relied on an in-person cadaveric dissection as a primary educational tool since the fifteenth century [4]. As opposed to other courses, anatomy requires an appreciation for complex three-dimensional relationships and is often one of first pre-clinical courses during which correlates to clinical medicine can begin to be illustrated. As such, a direct approach to “hands-on” anatomy education is perceived by some to be an indispensable component for subject mastery [5].

Previous studies have reported on the steady rate of modifications to US medical school anatomy education over the past two decades [6–9]. Such changes have included decreased total course time, decreased dissection time, and integration of anatomy education into other courses. However, no prior studies have included responses from more than 50% of medical schools in the US. Additionally, there have been no reports on the effects of the COVID-19 pandemic on US anatomy education or the future direction of the discipline’s pedagogy in the face of prolonged social distancing mandates. Therefore, we surveyed US medical schools to assess recent trends in anatomy education, the impact of the COVID-19 pandemic on anatomy teaching, and future anticipated directions of anatomy curricula.

## Methods

### Survey distribution

**Fig. 1:**
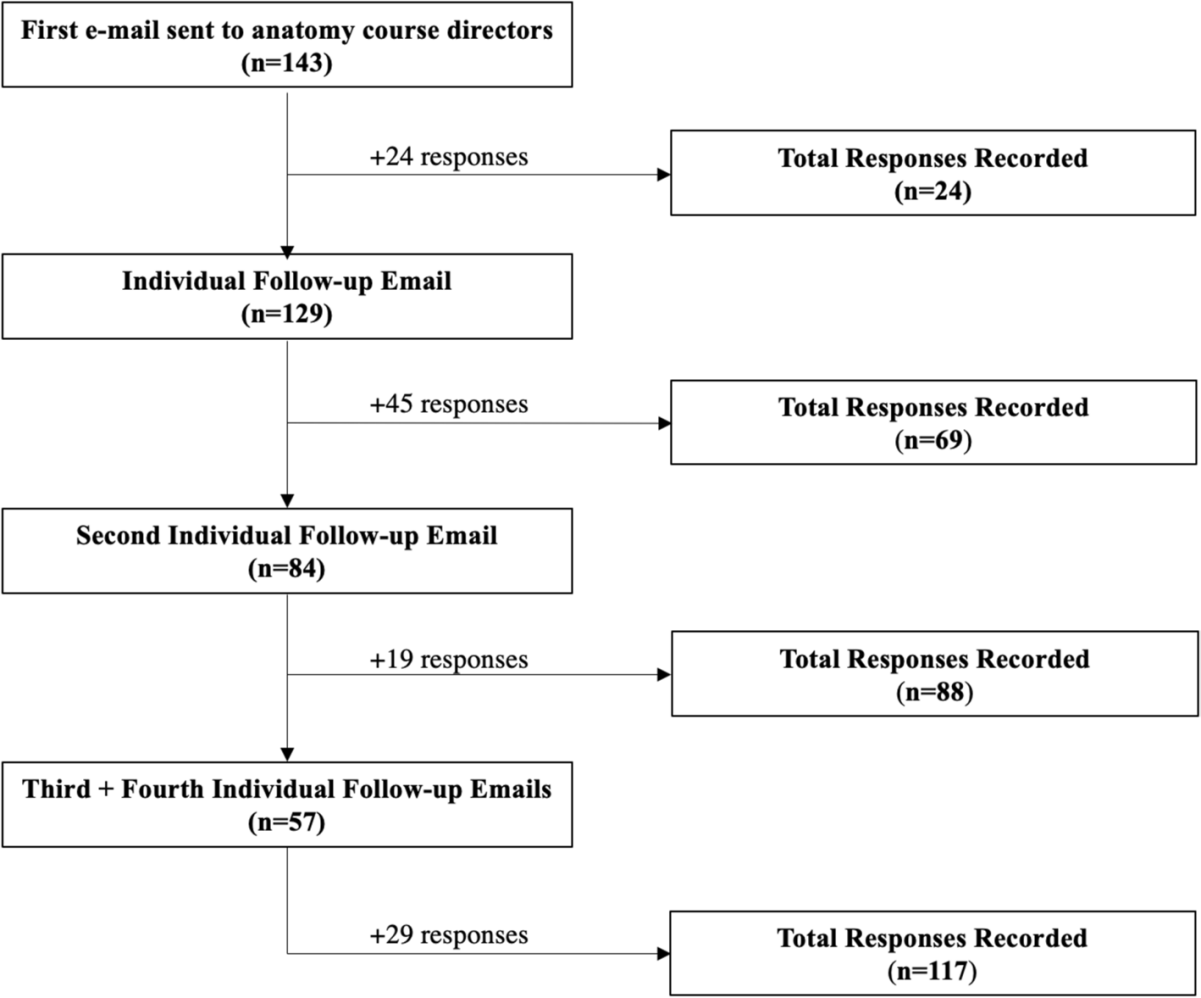
Flow diagram demonstrating survey response collection

All allopathic schools that were participating members of the Association of American Medical Colleges (AAMC) were identified. E-mail addresses for each school’s anatomy course director(s) were identified by searching faculty websites, Google search, or directly contacting the school’s medical education office. If multiple course directors were listed, e-mail addresses for all directors were included in the initial outreach. All collected addresses were then e-mailed a 29-item survey (Additional file 1) asking questions about their school’s gross anatomy curricula. Open-ended response questions also provided an opportunity to discuss the most recent changes to the school’s anatomy curriculum as well as any anticipated future changes. If no response was provided within a week, anatomy professors at each institution were individually e-mailed for follow-up. This was repeated three times for a total of four follow-up attempts (Fig. 1).

### Survey components

The distributed survey (Additional file 1) consisted of objective and subjective questions about each school’s gross anatomy curriculum. The first portion of the survey asked multiple-choice questions specific to each school’s gross anatomy curriculum before and during COVID-19. These included questions regarding course structure, teaching modalities, practical or “hands-on” learning (e.g. cadaver dissection/prosection, 3D/VR software, small group learning, etc.), use of supplemental material, and grading schemata. Respondents were also asked about their opinions of the effect of COVID-19 on the quality of their students’ anatomy education using a 5-point Likert scale. The last portion of the survey asked open-ended questions about curricula weaknesses, recent major curricula changes, and any anticipated future changes. Subjective responses were categorized into groups for analysis, as agreed upon by 2 authors (MS, AP).

### Statistical methods

Parametric and nonparametric continuous variables were summarized using mean and standard deviation or median and quartiles. Differences in parametric continuous variables between pre-COVID-19 and COVID-19 periods were assessed using Student’s T-Test. Non-parametric differences were assessed using the Wilcoxon Rank-Sum test. Chi-square analysis and Fisher’s Exact Test were used to assess the association between categorical variables. A two-sided Type I error rate of 0.05 was used to indicate statistical significance. All calculations were performed using STATA 14.2 (STATA Corp, College Station TX, USA).

## Results

Surveys were sent to one or more course directors or anatomy professors at 143 of 145 AAMC (98.6%) allopathic medical schools. Contact information was not available for the remaining two schools. A total of 117 (81.8%) responses were recorded. Among those that responded, 60 (51.3%) institutions taught gross anatomy within organ-systems blocks, while 54 (46%) taught anatomy as its own course or within a pre-organ system block (Table 1).

**Table 1:** Anatomy Course Characteristics (Pre-COVID-19)

Course Characteristic	*N* (%)
**Course Structure**	
Taught concurrently with organ-system blocks	60 (51.3)
Taught as own course or within pre-organ-systems blocks	54 (46.2)
Other	3 (2.5)
**Didactic Format**	
Lectures Live & Recorded	94 (80.3)
Lectures Live & Not Recorded	9 (7.7)
Lectures Pre-Recorded	31 (27.0)
Pre-Readings	17 (19.3)
Flipped Classroom	19 (16.2)
**Interactive Learning Format**	
Cadaver Dissection	106 (90.6)
Number of students per cadaver	5.1 ± 1.41
Cadaver Prosection	58 (49.6)
Virtual Software (e.g., Holo-Lens, 3D anatomy software, etc.)	13 (11.1)
Small-Group/Team-Based Learning	34 (29.1)
**Supplemental Resources**	
External Online Resources	77 (65.8)
Anatomy applications for phone, tablet, computer	75 (64.1)
In-House Resources	99 (84.6)
Other Resources	6 (2.3)
**Assessment**	
In-House Exams/Quizzes	107 (91.5)
NBME Exams	42 (35.9)
In-Person Practical	94 (80.3)
Online Practical	16 (13.7)
Standardized Patients	11 (9.4)

*NBME* ,  *National Board of Medical Examiners*

### Changes to anatomy curricula prior to COVID-19

Prior to COVID -19, the majority (*n* = 94; 80.3%) of institutions delivered didactics through live and recorded lectures. Nineteen (16.2%) institutions implemented a “flipped-classroom” approach to didactic learning. Cadaveric dissection (*n* = 106; 90.6%) was the most popular form of “hands-on” interactive learning, with an average of 5.1 ± 1.41 students assigned to each cadaver. Thirteen schools (11.1%) reported the use of novel virtual software (e.g. Holo-Lens, 3D virtual reality software, etc.) as a primary means of interactive learning, although 75 (64.1%) of schools provided anatomy applications to their students as a supplemental resource. Most schools (*n* = 65; 57%) reported a major change to their anatomy course within the past five years prior to COVID-19 (Table 2). Decreased total course time (19.7%), integration into other courses (18.8%), and implementation of flipped classroom in lieu of previous didactics (14.5%) were the most frequently reported changes. Among those course directors who reported a weakness of their course, answers centered around insufficient dissection time (23.1%) and total course time (15.4%) were most common.

**Table 2:** Recent and Anticipated Changes to Anatomy Curriculum

Feature/Question	*N* (%)
**Most Recent Major Change**	
< 5 years ago	65 (57.0)
5–10 years ago	24 (21.1)
10–15 years ago	9 (7.9)
15 + years ago	8 (7.0)
Unknown	11 (9.4)
**Last Recent Major Change (detail)**	
Implementation of Flipped Classroom	17 (14.5)
Anatomy integrated into other courses	22 (18.8)
Time Compression	23 (19.7)
Less time for (or elimination of) Dissection	16 (13.7)
Addition of Clinical Correlations/Radiology	11 (9.4)
Other	10 (8.5)
**Perceived Weakest Aspect of Course (Pre-Covid)**	
No apparent weaknesses/Not Reported	29 (24.8)
Insufficient time dedicated to dissection	27 (23.1)
Insufficient total course time	18 (15.4)
Course too fragmented	5 (4.3)
Insufficient Imaging (Ultrasound, X-Ray, CT Scan Interpretation)	5 (4.3)
Quantity/Quality of lab instructors	4 (3.4)
Time wasted on dissection	3 (2.6)
Time wasted on lecture	3 (2.6)
Insufficient Clinical Correlation	2 (2.3)
Other	13 (11.1)
**Future Changes Anticipated**	
None	43 (36.7)
Incorporation of Virtual Reality/3D Learning	27 (23.1)
Course Integration	6 (5.1)
Less Dissection	15 (12.8)
Further emphasis on radiology/CC	4 (3.4)
Flipped Classroom	8 (6.8)
Other	9 (7.7)

### Effect of COVID-19 on anatomy curricula

During COVID-19, online cadaveric prosection (students are provided with images of a cadaver which was previously dissected by an experienced anatomist) was the most common means of interactive learning (*n* = 50; 42.7%), and 28 (23.9%) schools reporting switching from cadaver dissection to prosection (Table 3). The majority of course directors (*n* = 78; 68.4%) indicated intentions to revert back to their pre-COVID curriculum structure following easing of pandemic related social distancing mandates.

**Table 3:** Anatomy course characteristics during COVID-19

Characteristic	*N* (%)
**Component of course *in person* this year**	
All Online	26 (22.2)
Lecture	18 (15.4)
Small-Group/Team-Based Learning	22 (18.8)
Cadaver Dissection	60 (51.3)
Cadaver Prosection	47 (40.7)
**Changes to ‘Hands-On’ Learning**	
No changes	12 (10.3)
Switch from dissection to prosection	28 (23.9)
Virtual/Online Prosection (i.e. showing images)	50 (42.7)
Use of Virtual Software	47 (40.2)
No Hands-On Learning	4 (3.4)
**Plan to return to previous course structure after Covid-19?**	
Yes	78 (68.4)
No	18 (15.8)
Uncertain	18 (15.8)

We found that COVID-19 has led to a significant decrease (*p* < 0.01) in both the weekly hours and the fraction of the course devoted to “hands-on” interactive learning (Table 3A). Due to COVID-19, the majority of schools (*n* = 62; 53.5%) used a Pass/Fail rubric with no internal relative performance ranking. Moreover, there was a significant decrease in the teaching of clinical correlates in anatomy courses (*n* = 100 [86%] vs *n* = 116 [99%]; *p* = 0.02) and imaging (*n* = 97 [83%] vs *n* = 109 [93.2]; *p* < 0.01).

**Table 3A:** Quantitative Effects of COVID-19 on Anatomy Education

Time	Pre-Covid-19	During Covid-19	*P*-Value
**Time / Distribution**			
Weekly Hours of Lecture	4.3 ± 3.0	4.38 ± 3.1	0.63
Weekly Hours of Active Learning	6.2 ± 3.3	4.2 ± 3.1	< 0.001
% Lecture	38.2 ± 17.6	44.3 ± 23.2	0.001
% Active Learning	61.8 ± 17.6	55.7 ± 23.2	0.001
**Grading Scheme**			0.001
Pass/Fail no Ranking	56 (48.7)	62 (53.5)	
Pass/Fail with Ranking	33 (28.7)	29 (25.0)	
Graded (letters)	13 (11.3)	11 (9.5)	
Honors, High Pass, Pass, Fail	9 (7.8)	8 (6.9)	
Unclear	4 (3.5)	6 (5.2)	
**Teaching of Radiology/Imaging**	109 (93.2)	97 (82.9)	< 0.001
**Teaching of Clinical Correlates**	116 (99.2)	100 (85.5)	0.02

When course directors were asked to compare students’ performances on assessments during COVID-19 to those of previous years (Table 3B), the most common response was ‘The Same’ (*n* = 63; 53.9%). However, when they were asked about their opinion of the effect of COVID-19 on the quality of anatomy education, ninety-two respondents (78.6%) reported ‘Slight’ or ‘Significant Negative Impacts’. Among those reporting negative effects, ‘Less time devoted to interactive learning’ (62.4%), ‘Less time learning in-person’ (62.4%), anxiety (59.0%), and ‘Lack of Dissection’ (56%) were the most cited justifications.

**Table 3B:** Qualitative Effects of COVID-19 on Anatomy Education

**Feature/Question**	*N* (%)
**Student Performance**	
Significantly Worse	3 (2.6)
Slightly Worse	16 (13.7)
The Same	63 (53.9)
Slightly Better	29 (24.8)
Significantly Better	6 (5.1)
**Quality of Learning**	
Significant Negative Impact	22 (18.8)
Slight Negative Impact	70 (59.8)
No Impact	13 (11.1)
Slight Positive Impact	10 (8.6)
Significant Positive Impact	2 (1.7)
**Reasons for ‘Slight Negative’ or ‘Significant Impact’ (*N* = 71)**	
Anxiety	69 (59.0)
Less time spent in-person learning	73 (62.4)
Inferior online curriculum	51 (43.6)
Less time devoted to interactive learning	73 (62.4)
Lack of Dissection	52 (44.4)
Lack of Prosection	19 (16.2)
Less time overall for Anatomy	20 (17.1)
Disorganization	13 (11.1)
**Reasons for ‘Slight Positive’ or ‘Significant Positive Impact’ (*N* = 12)**	
Time saved from eliminating dissections	3 (25.0)
More time devoted to practical learning	4 (33.3)
Superior online curriculum	4 (33.3)
Adoption of 3D/Virtual-Reality dissection	1 (8.3)

### Anticipated changes to anatomy structure & curriculum

Lastly, answers pertaining to the incorporation of virtual-reality software or novel 3D learning platforms (23.1%) and reducing time spent on cadaver dissection (12.8%) were the most commonly reported anticipated future changes among institutions planning to institute a change.

## Discussion

To our knowledge, this is the first study to describe the current state and future of medical school gross anatomy education with over 80% course director participation. It is also the first study to objectively and subjectively analyze the impact of COVID-19 and how this impact fits within recent trends in US medical school anatomy education. While we found a continuation of general educational trends described by previous authors [7, 9], we also report on recent changes in didactic approaches and novel future directions for anatomy education, potentially catalyzed by social distancing mandates imposed by COVID-19.

In accordance with prior work, we found that a growing number of institutions have integrated anatomy education into organ-system blocks. Cadaveric dissection remained the most popular mode of interactive learning among course directors, and our study found the proportion of schools using dissection (90%) prior to COVID-19 (2018–2019) to be similar to that reported by a similar study assessing the 2016–2017 year [9]. We also found that a majority of medical schools provided some form of supplemental external online resource, including phone or table applications, for their students to use as a supplement to traditional lectures and coursework. Interestingly, our survey results indicate that some schools do not utilize practical learning as a form of formalized assessment. We found that 78.4% make use of in-person practical exams, while 13.6% use virtual practical exams. By extension, this implies that a minimum of 8% of schools do not use any form of practical evaluation of knowledge, despite previous literature assessing its efficacy as a summative assessment tool [10]. A small proportion of institutions also incorporate standardized patients into their student performance assessments, which may be of particular use in developing students’ competencies beyond the application of anatomy knowledge.

Our study also sought to examine recent major changes to US anatomy curricula prior to COVID-19. In addition to a compression of course hours and integration of anatomy into other courses – which have been previously reported on [7, 9] – we found that many institutions have recently adopted, or plan to adopt, a ‘flipped classroom’ approach to learning, wherein students independently gain an understanding of material, allowing greater class time to be devoted to application and discussion [11]. A recent meta-analysis examining the flipped classroom approach in healthcare professional education courses, including anatomy, concluded that flipped-classroom approaches to learning were preferred by students and resulted in increased learning performance [12]. The authors attributed these findings to increased temporal flexibility in synthesizing material and—importantly—to an increase in the amount of active learning afforded by the lecture time saved. Flipped classroom teaching modalities may be especially pertinent for anatomy education, given our study’s findings indicate that the most common weakness of anatomy curriculum as reported by anatomy directors is insufficient dissection time, which may be considered a form of active learning. Furthermore, a lack of time devoted to practical and in-person learning were the most cited reasons for the pandemic’s negative impact on anatomy education. These findings are logical, as anatomy requires an understanding of three-dimensional relationships that may be appreciated through cadaveric dissection but may be difficult to capture through two-dimensional media, such as lecture slides or textbooks. Utilization of a flipped classroom approach may be a prudent future direction for anatomy education as it will allow educators to maximize formalized curriculum time spent on interactive or in-person learning.

The COVID-19 pandemic has dramatically affected the landscape of medical education [13]. While it was admittedly commonplace for students to forego in-person preclinical lectures prior to the pandemic [14], the loss of those aspects of medical education that require collaboration and physical presence have and will continue to detract from the learning experience and student engagement. Furthermore, beyond being tasked with revamping an entire curriculum seemingly overnight, medical educators, often physicians themselves, have the added responsibility of remaining at the frontline of patient care during the pandemic. Thus, in assessing the effects of COVID-19 on anatomy education, we were unsurprised to find that a majority of anatomy course directors found the COVID-19 pandemic to have a slight or significant negative impact on the quality of learning due to a reduction in practical and in-person learning. Specifically, social distancing mandates tended to lead to an increase in the fraction of course time devoted to lecture, with a corresponding decrease in the amount of active learning time. Interestingly, however, most course directors indicated that student performance on assessments did not change. This can likely be explained in part due to changes in how student assessments were conducted during COVID-19. Prior to COVID-19, 78% of schools reported the use of in-person practical exams as part of their assessment. In contrast, during COVID-19, 25% of course directors reported a completely virtual curricula this year. The lack of an in-person cadaveric practical exam may in part explain these findings, as students may not have needed to demonstrate a proficiency in three-dimensional relationships of the body, but rather memorize images that appeared on virtual assessments. These findings highlight the importance of interactive and practical application-based education in learning complex relational subjects such as anatomy. While the majority of surveyed institutions intended to return to their pre-COVID-19 course curriculum following the pandemic, 16% indicated otherwise, potentially reflecting permanent adoption of new educational tools developed or acquired as a result of the pandemic.

Interestingly, we found a significant decrease in the number of schools that taught clinical anatomy correlates and radiology during this period, which have previously been linked to significant enrichment in student knowledge [15, 16]. These findings could arise from a few possible explanations: the sudden-onset nature of the pandemic amidst the school-year forced educators to immediately transition entire courses to an online-format, which may have led to holes in curricula. Physician-educators who teach clinical correlates and imaging may have found themselves burdened with new or additional responsibilities during this time. Additionally, there has been significant incorporation of ultrasound teaching during anatomy courses in previous years [17]. Thus, though one would expect a transition to online learning to have no effect on radiological teaching, a decrease in ultrasound pedagogy, owing to its traditional in-person setting, could explain these findings.

Looking ahead at anticipated future changes to US anatomy education, it appears there will be a growing movement away from time dedicated to dissection as well as an embracement of virtual-reality software. In this light, the COVID-19 pandemic has further highlighted the need to leverage modern technologies to improve efficiency in anatomy education [18, 19]. While decreases in dedicated cadaver dissection time has been a well-recognized trend in recent years [8, 20], we found that 23% of institutions planned on incorporating virtual software/mixed-reality learning into their pedagogical armamentarium in the near future. In certain ways, this may reflect one of the few benefits to medical education spurred upon by the COVID-19 pandemic, as a recent article examining the use of mixed-reality technologies during the pandemic found it to be an effective method of learning anatomy with advantages over traditional approaches [21]. Similar findings have also been shown in a previous meta-analysis [22]. Furthermore, the cost of obtaining, storing, and appropriately caring for cadavers can also be costly, especially during the COVID-19 era during which numerous institutions have taken the precautionary step of ceasing acceptance of cadaver donations. Virtual educational tools may help account for such shortages and decrease costs associated with conducting anatomy education. While virtual dissection as a supplement to traditional cadaveric dissection appears to be a promising direction for anatomy education, our findings that most course directors intend to revert back to their pre-COVID curriculum indicate that virtual software, in its current form, is an insufficient substitute for cadaveric dissection. Thus, an increased emphasis on virtual learning should be incorporated with caution to ensure there are no negative tradeoffs in education with this approach.

### Limitations

This study had several limitations. First, we were unable to collect responses from 19% of institutions, and there are medical schools in the US beyond those that are members of the AAMC, most notably osteopathic institutions. Thus, our findings may not be fully reflective of anatomy education in the US at large. However, to our knowledge, our response rate of 80% is the highest among similar survey-based studies in anatomy education. Our survey asked about the weekly time commitment of didactics and interactive learning, and we did not ask about total course hours, which could have provided a useful metric. Previous authors have noted calculating total course hours for an anatomy course to be laborious for course directors to estimate, especially for those in integrated curriculums, and a potential reason for their low response rates [9]. Thus, we additionally asked course directors to estimate the relative split between time dedicated to lecture and interactive learning. Furthermore, our survey did not include questions about the course directors themselves, including age, experience, and educational background. Differences across these factors could lead to differences in opinion and should be considered in future studies. Lastly, the COVID-related subjective questions were answered by the course director of each institution, which may be biased by personal opinion and not necessarily reflective of students’ learning experiences. While a more comprehensive survey would also consider student experiences, many students would not have a non-COVID era anatomy course to compare their experience to, and thus we decided that course directors who inherently have a more longitudinal perspective would be most appropriate to survey.

## Conclusion

Our study highlights the state of anatomy medical education in the United States during immediate pre- and mid-COVID-19 time points, characterizes adaptations made to accommodate the pandemic, and reports on potential directions of future curricula. We found an increasing adoption of new approaches to didactics and online interactive learning modalities that may be appropriate substitutions for traditional methods in some cases. Lastly, our analysis of course director experiences and opinions indicate the importance of maximizing interactive learning during a period in which anatomy course time has been decreasing.

## Supplementary Information

**Additional file 1.** Survey Instrument. 

## Data Availability

The data for this project were the results from a survey sent to anatomy professors across the United States. We would prefer not to make this data publicly accessible; while the institution names can be omitted, we feel that individual institutions may still be identifiable based on their responses. Additionally, we asked several questions in our survey that asked for subjective opinions and would like to maintain their privacy. No publicly available data was used for this project. Should someone request the data, they can contact Max Shin.

## Abbreviation

COVID-19: Coronavirus 2019
