# Supplementary material for: Anatomy education in US Medical Schools: before, during, and beyond COVID-19
Source: BMC Med Educ. 2022 Feb 16;22:103. doi: 10.1186/s12909-022-03177-1 (PMC8851737; doi:10.1186/s12909-022-03177-1)
Supplement: Supplementary file 1 — Additional file 1. Survey Instrument. [file 12909_2022_3177_MOESM1_ESM.pdf]

Anatomy Changes during COVID

We are incredibly appreciative of your time. The survey below should not take more than 5 minutes and will be incredibly informative to the current state of anatomy education.

Please also note that any questions that begin with [PRE-COVID] pertain to your anatomy course PRIOR to COVID-19

Conversely, any questions that starts with [THIS YEAR DURING COVID] pertain specifically to the most recent cohort of students who took/are taking anatomy DURING the COVID-19 pandemic

Lastly, ALL REPORTED DATA WILL BE COMPLETELY ANONYMOUS and RESULTS WILL NOT BE ABLE TO BE TRACED BACK TO YOU OR YOUR INSTITUTION.

Please state the medical school you teach at (Note: All reported data will be anonymous)

____

[PRE-COVID] How is your main anatomy curriculum usually structured?

◯ Taught alongside organ-system blocks
◯ Taught as its own course (e.g. 12 weeks in a row)
◯ Other

Please describe if "other"

[PRE-COVID] Please describe how your anatomy lectures/didactics are usually delivered (please check all that apply)

☐ Lectures are given live and ARE NOT recorded
☐ Lectures are given live and ARE recorded
☐ Lectures are pre-recorded
☐ Pre-Readings
☐ Other

Please describe if "other"

[PRE-COVID] How do students usually learn during the 'anatomy lab' (non-didactic) portion of your curriculum? Please check all that apply

☐ Cadaver Dissection
☐ Cadaver Prosection
☐ Virtual (i.e. Holo-Lens, 3D anatomy software)
☐ Small Group / Team-based Learning
☐ Other

If dissection, please indicate the average number of students assigned to each cadaver

____

Please describe if "other"

[THIS YEAR DURING COVID-19] Was any part of your anatomy curriculum in-person this year? We will assume all activities were socially-distanced. Please check all that apply

☐ No portion of the curriculum was in-person
☐ Lectures
☐ Small-group/team-based learning
☐ Cadaver prosection
☐ Cadaver dissection
☐ Other forms of practical learning

Please describe if "other forms of practical learning"

[THIS YEAR DURING COVID] In response to the pandemic, how did you adapt your lab-based learning this year? Please check all that apply

☐ No change
☐ Switch from dissection to prosection
☐ Virtual prosection (e.g. showing images)
☐ Use of Virtual application (anatomy applications, 3D software, etc.)
☐ There was no lab-based component this year
☐ Other

Please describe if "other"

[PRE-COVID] During an average week of the course, how many hours of anatomy lecture are students roughly expected to attend/watch?

____

[THIS YEAR DURING COVID] During an average week of the course THIS YEAR, how many hours of anatomy lecture were students roughly expected to attend/watch?

____

[PRE-COVID] During an average week of the course, how many hours do students usually spend participating in practical learning (Anatomy Lab or its equivalent)

____

[THIS YEAR DURING COVID] During an average week of the course THIS YEAR, how many hours did students spend participating in practical learning (Anatomy Lab or its equivalent)

____

[PRE-COVID] Overall, how is time split between didactic learning (lectures) vs. practical learning (lab, small groups, Team-based Learning, etc.)

0% Lecture; 100% Practical	50% Lecture; 50% practical	100% Lecture; 0% Practical

(Place a mark on the scale above)

[THIS YEAR DURING COVID] Overall, how was time split between didactic learning (lectures) vs. practical learning (lab, small groups, Team-based Learning, etc.) this year?

0% Lecture; 100% Practical | 50% Lecture; 50% Practical | 100% Lecture; 0% Practical

(Place a mark on the scale above)

[PRE-COVID] Is radiology (interpretation of X-ray, MRI, CT, etc.) taught alongside your anatomy curriculum?

◯ Yes
◯ No

Did this change this year due to the pandemic?

◯ Yes
◯ No

[PRE-COVID] Are clinical correlates (i.e. applications of anatomy to clinical scenarios/vignettes) taught in your anatomy curriculum?

◯ Yes
◯ No

Did this change this year due to the pandemic?

◯ Yes
◯ No

What supplementary materials do you provide to students? (please check all that apply)

☐ None
☐ External Online resources
☐ Anatomy Apps (for tablets, computers, phone)
☐ In-house resources (i.e. guides, e-books, etc.)
☐ Other

Please describe if "other"

[PRE-COVID] What is the format usually used for assessment of students? (Please check all that apply)

☐ In-house quizzes/exams
☐ NBME Exams
☐ In-person Lab Practical
☐ Virtual Practical
☐ Standardized Patients
☐ Other

Please describe if "other"

____

How did this year's students (during COVID-19) perform on assessments as compared to previous years (prior to COVID-19)?

◯ Significantly worse
◯ Slightly worse
◯ The same
◯ Slightly better
◯ Significantly better

[PRE-COVID] Please describe the overall grading schema for your anatomy course

◯ Pass/Fail with no internal ranking
◯ Pass/Fail with external or internal ranking
◯ Honors, High-Pass, Pass, Fail
◯ Graded (Letters)
◯ I don't know

[THIS YEAR DURING COVID] Please describe the overall grading schema for your anatomy course

◯ Pass/Fail with no internal ranking
◯ Pass/Fail with external or internal ranking
◯ Honors, High-Pass, Pass, Fail
◯ Graded (Letters)
◯ I don't know

How do you think COVID-19 affected the quality of anatomy education this year at your institution?

◯ Significant negative impact
◯ Slight negative impact
◯ No impact
◯ Slight positive impact
◯ Significant positive impact

Please check all of the following that you believe contributed to this negative impact

☐ Anxiety related to the pandemic
☐ Lack of in-person learning
☐ Virtual learning less conducive
☐ Less time spent in practical learning/lab
☐ Lack of cadaver dissection
☐ Lack of cadaver prosection
☐ Overall less structured time for anatomy
☐ Disorganization
☐ Other

Please describe if "other"

Please check all of the following that you believe contributed to this positive impact

☐ Time saved from eliminating dissection
☐ Students learned better from virtual curriculum
☐ More time spent in practical learning
☐ Other

Please describe if "other"

Prior to COVID-19, what did you perceive to be the weakest part of your anatomy course?

Prior to COVID-19, when was the last major change to your anatomy curriculum? Examples include switching from dissection to prosection, switching to virtual platforms, flipped classroom, etc.

◯ < 5 years ago
◯ 5-10 years ago
◯ 10-15 years ago
◯ 15+ years ago
◯ Do not know

If possible, please specify what the change was

Are you anticipating any changes in the near future to your anatomy course (e.g. adoption of virtual software, transitioning to or from dissection/prosection)? If so, please briefly describe

Once social distancing is no longer required, do you expect your anatomy curriculum/structure to return to how it was prior to the pandemic?

◯ Yes
◯ No
◯ I don't know

If you have any additional thoughts you would like to share about how COVID-19 impacted your anatomy curriculum or your school's adaptations, please share here
